# Biomimetic and biodegradable cellulose acetate scaffolds loaded with dexamethasone for bone implants

**DOI:** 10.3762/bjnano.9.189

**Published:** 2018-07-13

**Authors:** Aikaterini-Rafailia Tsiapla, Varvara Karagkiozaki, Veroniki Bakola, Foteini Pappa, Panagiota Gkertsiou, Eleni Pavlidou, Stergios Logothetidis

**Affiliations:** 1 Lab for “Thin Films - Nanosystems & Nanometrology”, Nanomedicine Group, Department of Physics, Aristotle University of Thessaloniki, Greece; 2 BL NanoBiomed P.C. Thessaloniki, Greece; 3 Department of Physics, Aristotle University of Thessaloniki, Greece

**Keywords:** drug delivery, electrospinning, nanocoatings, orthopedics, tissue engineering

## Abstract

There is, as a matter of fact, an ever increasing number of patients requiring total hip replacement (Pabinger, C.; Geissler, A. *Osteoarthritis Cartilage* **2014,** *22,* 734–741). Implant-associated acute inflammations after an invasive orthopedic surgery are one of the major causes of implant failure. In addition, there are instability, aseptic loosening, infection, metallosis and fracture (Melvin, J. S.; Karthikeyan, T.; Cope, R.; Fehring, T. K. *J. Arthroplasty* **2014,** *29,* 1285–1288). In this work, a drug-delivery nanoplatform system consisting of polymeric celluloce acetate (CA) scaffolds loaded with dexamethasone was fabricated through electrospinning. Atomic force microscopy (AFM) and scanning electron microscopy (SEM) indicated the successful fabrication of these structures. Cytotoxicity studies were performed by using MTT assay, methylene-blue staining and SEM fixation and showed very good cell adhesion and proliferation, indicating the cytocompatibility of these fibrous scaffolds. Drug-release kinetics was measured for the evaluation of a controllable and sustained release of anti-inflammatory drug onto the engineered implants and degradation study was conducted in order to assess the mass loss of polymers. This drug-delivery nanoplatform as coating on titanium implants may be a promising approach not only to alleviate but also to prevent implant-associated acute inflammations along with a simultaneous controlled release of the drug.

## Introduction

The application of nanotechnology in medicine, known as nanomedicine, aims to overcome problems associated with diseases at the nanoscale, which is the size of the majority of biological molecules [1]. In nanomedicine, new drug-delivery systems can be designed to obtain, combined with state-of-the-art implantation technology, implants with therapeutic agents that are released at the site of implantation. The aforementioned systems are placed as coatings in medical devices in order to enhance the biocompatibility [2–4].

One technique to produce such coatings is electrospinning, which yields long micro- and nanofibers [5]. More specifically, physical and synthetic polymeric fibers of 30–20000 nm in length are produced by using an electrostatically charged jet of polymer solution [6]. It should be mentioned that such electrospun scaffolds are a very promising approach for the regeneration and repair of bones and related tissue [7] in total hip replacement or bone-fracture repair. The number of patients in need of such surgeries is envisaged to increase rapidly either as the people get older or due to car accidents [8–10]. All bone substitute materials must be bioactive and behave similarly to healthy bones [11]. There is a steadily increasing interest in natural, semi-synthetic and synthetic polymeric biomaterials as three-dimensional (3D) polymeric scaffolds acting as substrates for the growth, differentiation and proliferation of biological cells for their successful implementation in medical devices. This has led to the development of a new generation of diagnostic and therapeutic approaches [12–13]. Although synthetic polymeric scaffolds exhibit controllable degradation, good mechanical properties and can be modified easily, they cannot be as bioactive as natural polymers. Moreover, due to their hydrophobicity they hinder significant cellular growth and subsequent tissue formation [14].

Therefore, cellulose acetate (CA) is used in this work. CA is derived from a natural polymer and is biocompatible, biodegradable, nonirritant and nontoxic. Moreover, it has excellent mechanical properties and there are potential applications, for instance as films, membranes, tissue engineering scaffolds and drug-delivery devices [15–17]. The use of micro- and nanofibers as carriers for drug release is more efficient because the drug is locally released to the target organ or tissue and as a result less amount of drug is required with fewer side effects [18–19]. Inflammation is the most common cause of aseptic implant failure after a total hip replacement [20–21]. Long-term treatment with glucocorticoid drugs together with anti-inflammatory and immunosuppressive agents such as dexamethasone (dexam) is applied to face this challenge [22]. Non-woven CA nanofiber meshes drug-loaded with dexam were produced through electrospinning, in order to prevent the inflammation that can occur after a total hip replacement surgery.

## Experimental

### Materials and methods

#### Materials

Cellulose acetate (CA, *M*_w_ = 30,000 g/mol), dexamethasone (≥97%), acetone (≥99.8%), trypsin and methylene blue were all purchased from Sigma-Aldrich, Germany. *N*,*N*-Dimethylacetamide was obtained from Chem-Lab NV, Belgium. In MTT assay the cells used in this study were mice fibroblasts (L929) and phosphate-buffered saline (PBS), Dulbecco’s Modifies Eagle Medium (DMEM), 10% fetal bovine serum (FBS) and antibiotics were obtained from Gibco^®^ Cell Culture.

#### Preparation of polymer and drug solution

The CA solution (20%, w/w) was prepared by dissolving CA in a mixture of acetone and dimethylacetamide and stirred with a magnetic stirrer over night at room temperature. The dexamethasone solution was created by adding dexamethasone to acetone and placed in a Vortex apparatus until the drug is completely dissolved. Afterwards, the solution of dexamethasone was added to the CA solution for the subsequent use in the electrospinning process.

#### Electrospinning process

The ES-2000S electrospray deposition (ESD) equipment was sued to deposit the sample onto a substrate through electrostatic forces. The solution mentioned above was placed in a glass syringe with a metal needle and high voltage was applied between the needle and the collector. The high voltage produces an electrically charged jet of polymer solution, which dries and thus a polymer fiber is created [23]. Grounded aluminum foils, glass substrates and also coatings for orthopaedic pins were used as collectors to carry out the necessary studies.

#### Drug-release kinetics and degradation study of scaffolds

Degradation study was carried out in order to evaluate the mass loss of polymer and the changes in the macrostructure. The molecular weight of the polymer influences the degradation rate and the higher the molecular weight is, the lower the degradation rate becomes, because there is a greater number of ester bonds to be cleaved due to the bigger chain length [24]. The degradation study was examined in both pure CA and drug-loaded CA scaffolds over a period of 150 days to determine how the degradation rate is affected by the presence of the drug. Finally, release kinetics of drug-loaded CA electrospun scaffolds was measured.

**Degradation study:** First, the electrospun samples were cut as accurately as possible to the same size and placed in a 24-well plate. Subsequently, using an analytical balance (KERN & Sohn GmbH SEALED, ABT 120-5DM), each sample was weighed three times for repeatability. After that, 1 mL of DMEM was added to each sample followed by incubation at 37 °C (New Brunswick, Galaxy 170S). The samples remained in medium and after a chosen period of time (days), DMEM was removed from the samples, which were then exposed to air in a fume hood (Telstar Technologies, S.L. PV-30/70) until they had dried completely at room temperature. Finally, the samples were weighed again and the degree of degradation was determined according to the following equation:

[1]
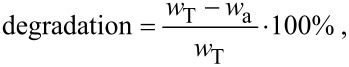


where *w*_a_ is the original weight of each sample before degradation, and *w*_T_ is the residual weight after degradation of the same samples and their complete drying.

**Drug-release kinetics:** A similar process was also performed for the drug release kinetics. The samples were placed into a 24-well plate, and then 2 mL of PBS was added to each sample and incubated at 37 °C in 5% CO_2_. The release of the drug was determined as a function of the time. For this, an aliquot of 100 μL was removed and the absorbance of the medium was measured at the absorption wavelength of the drug, using a 96-well plate reader (Luminometer Promega Glomax multi detection system). After that, the remaining samples were placed back in the incubator until the next measurement.

#### In vitro cytotoxicity assays

**MTT assay direct test and methylene blue staining:** L929 mouse fibroblasts were used to examine the cytotoxicity levels due to their properties and biological characteristics (biological responses and reproducible growth rates). The samples were placed inside a well-plate and 1 mL of medium was added and the whole system was left in the incubator for about 20 min, which is a sufficient time for the microenvironment to be generated. In the meantime, subculturing of the L929 cells was followed. The procedure was repeated at days 1, 2 and 5. The next step was PBS washes, trypsination and centrifuging at 5000 rpm for 5 min of the cells in order to create a cell pellet. The cell pellet was dissolved in DMEM and a quantity of 200 μL was put in every single well. Then, the well-plate was placed in the incubator for about 30–40 min. An amount of 1 mL of medium was added to every single well until the next day. The next day, it was removed and 500 μL of PBS was added.

Methylene blue is commonly used for the identification of cell viability as it stains the nuclei of living cells, making them more observable. The protocol for staining with methylene blue initially involves the addition of methanol to the samples, which were in the well-plates for 5 min*.* Then, after removing methanol, methylene blue was added for 30 min. After removing the methylene blue, the samples were rinsed with distilled water until the blue color disappeared. Finally, after removing the lid of the well-plate, the samples were exposed to room temperature in order to dry and to be subsequently used for SEM analysis.

## Results and Discussion

### Development of drug-free and dexamethasone-loaded CA scaffolds

**Figure 1:**
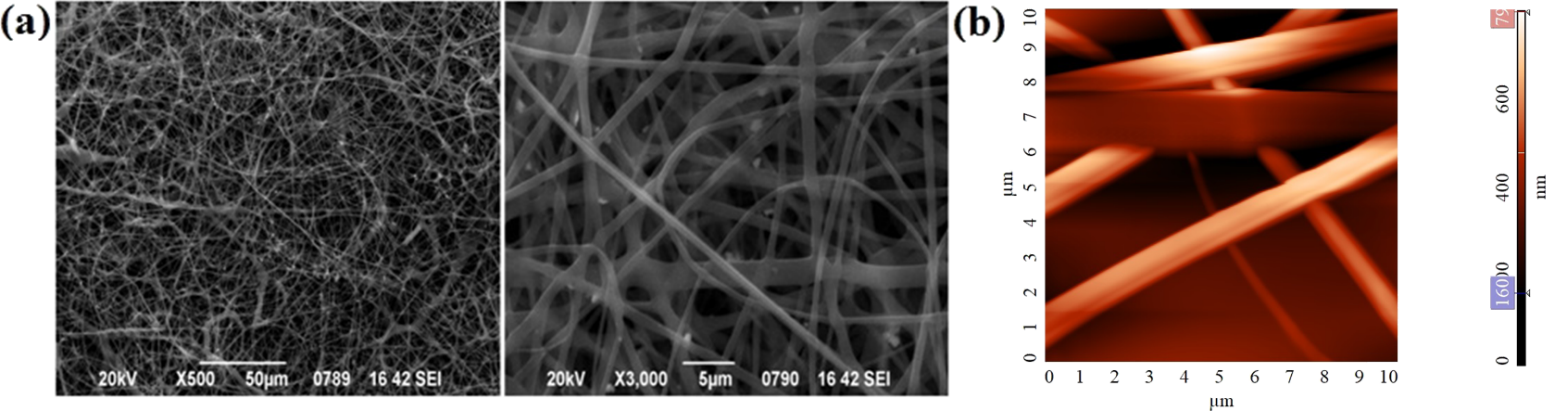
(a) Representative SEM micrographs of electrospun CA fibers, (b) AFM topography image of CA scaffolds with root mean square *S*_q_ = 135 nm and peak-to- peak *S*_y_ = 795 nm.

Fibers of drug-free CA and CA loaded with dexamethasone were created through electrospinning. SEM and AFM indicated the successful fabrication of those structures (Figure 1). Continuous fibers with smooth surface and free of any beads and other defects were obtained.

**Figure 2:**
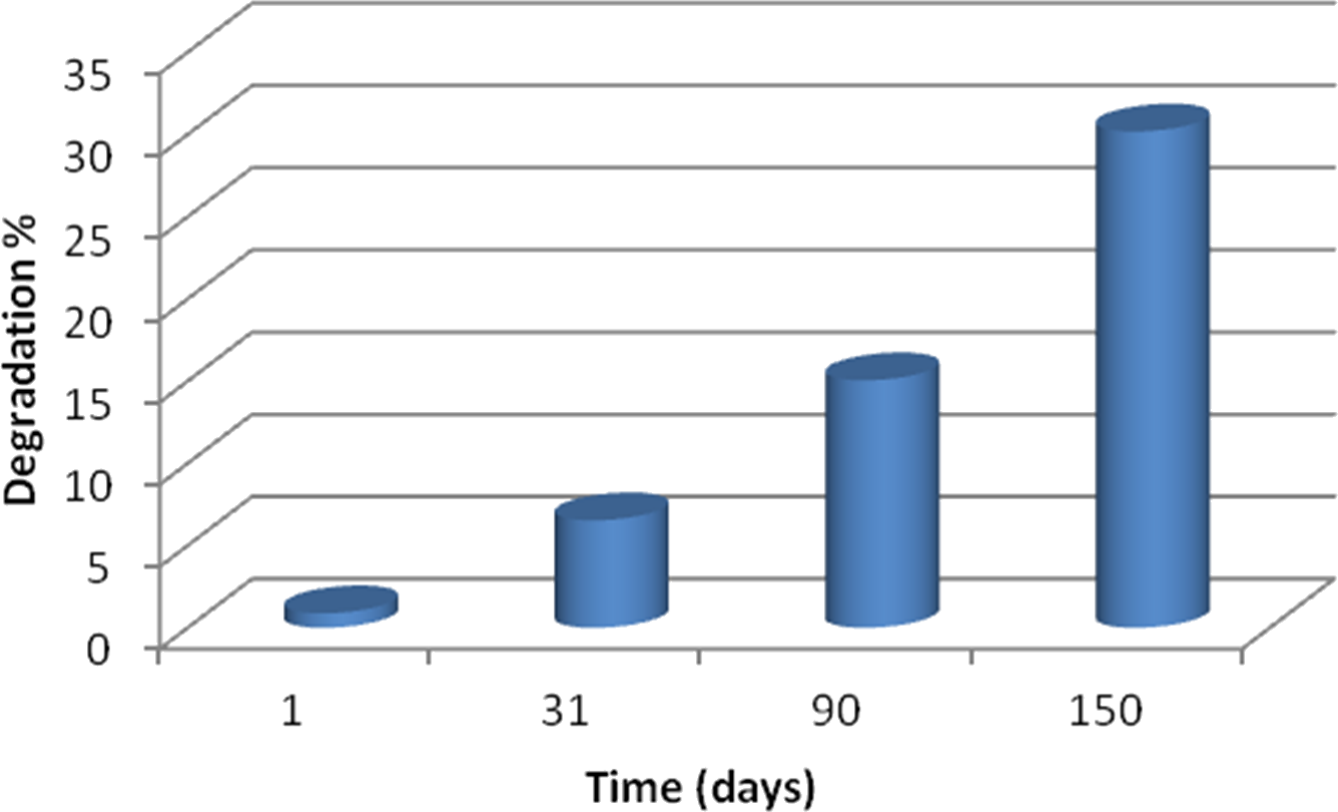
In vitro degradation of CA scaffolds as a function of the time.

In vitro degradation of non-woven CA fibers was investigated in DMEM solution at 37 °C over a period of 5 months (Figure 2). It should be mentioned that, CA is a semi-synthetic polymer, produced by the partial esterification of cellulose with acetic acid. The esterification of hydroxy groups of cellulose increases the hydrophobicity of CA while at the same time the existence of ester bonds makes it more susceptible to degrading in aquatic environments. So, in a 5 month period the change of the scaffold mass was measured and the results are presented in Figure 2. A slow degradation rate was observed, with only 30.2% degradation after 150 days. This can be attributed probably to the polymer structure, its molecular weight and other characteristics.

**Figure 3:**
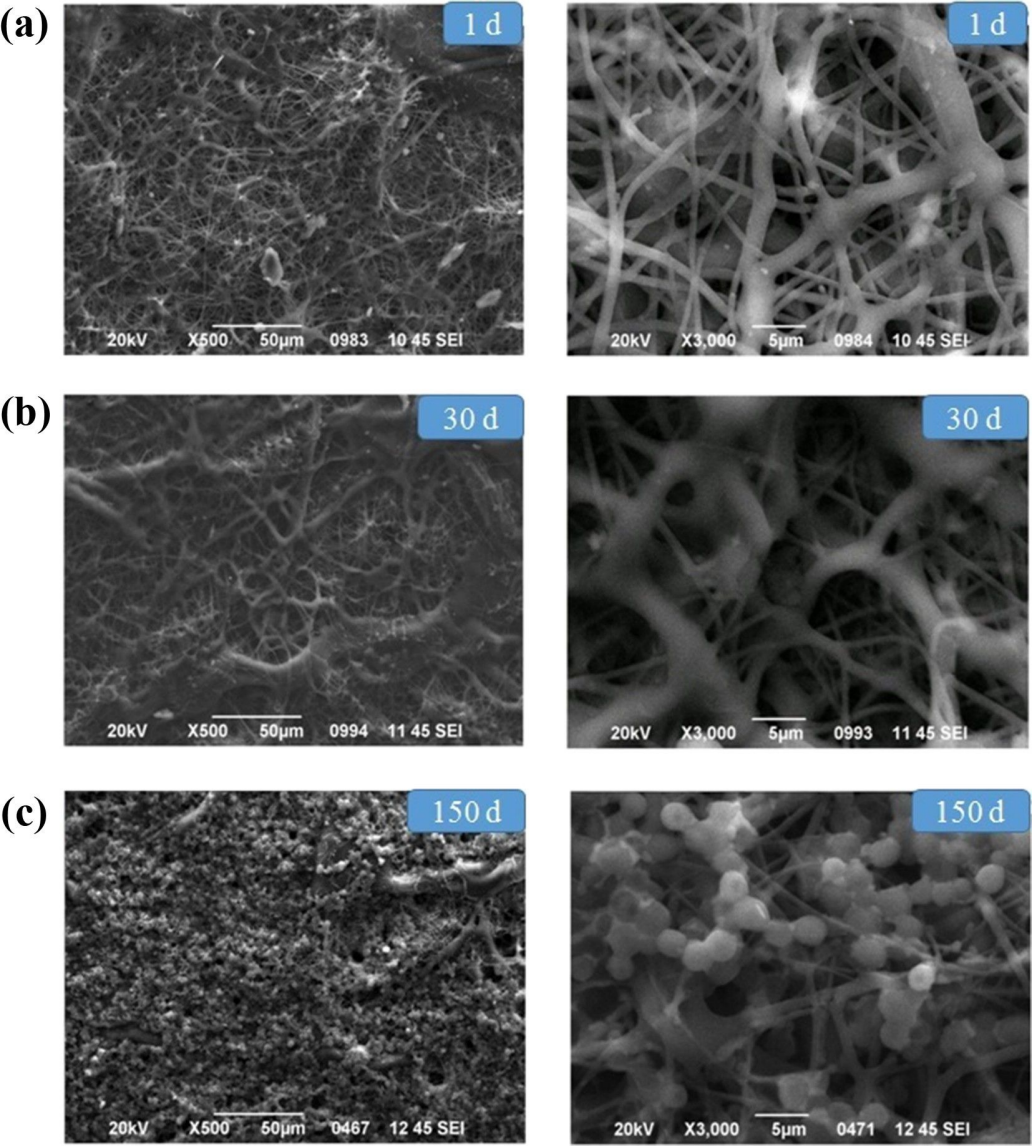
Representative SEM micrographs of CA scaffolds (a) before degradation on the 1st day, (b) after degradation in vitro for 30 days, (c) after degradation in vitro for 150 days. The mean diameters of the CA fibers after day 1 and day 30 were 1040 and 2400 nm, respectively.

The changes in molecular weight of the polymer and the degradation of CA fibers were studied via SEM after 1, 30 and 150 days (Figure 3). 20 fibers were randomly selected from the images at the highest magnification (3000×) and their diameter was determined by using the ImageJ software. A swelling of fibers was observed after day 30, as confirmed by the increase of their diameter (from 1040 to 2400 nm). After 150 days, the polymeric fibers that were on top of the surface had been degraded to a great extent, making it impossible to calculate their diameter.

**Figure 4:**
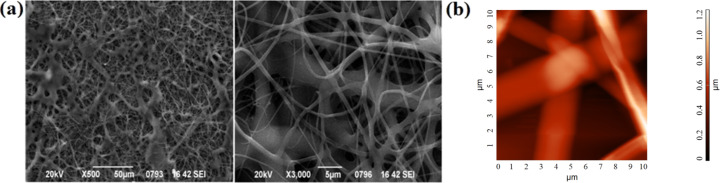
(a) Representative SEM micrograph of electrospun CA:dexam fibers, (b) AFM topography image of CA:dexam scaffolds with root mean square *S*_q_ = 206 nm and peak-to-peak *S*_y_ = 1203 nm.

Afterwards, the fabrication of CA scaffolds loaded with dexamethasone was investigated through electrospinning. Optimized electrospinning conditions were found and the characterization of those fibers via SEM and AFM showed that a fiber morphology without beads and other defects was achieved (Figure 4).

**Figure 5:**
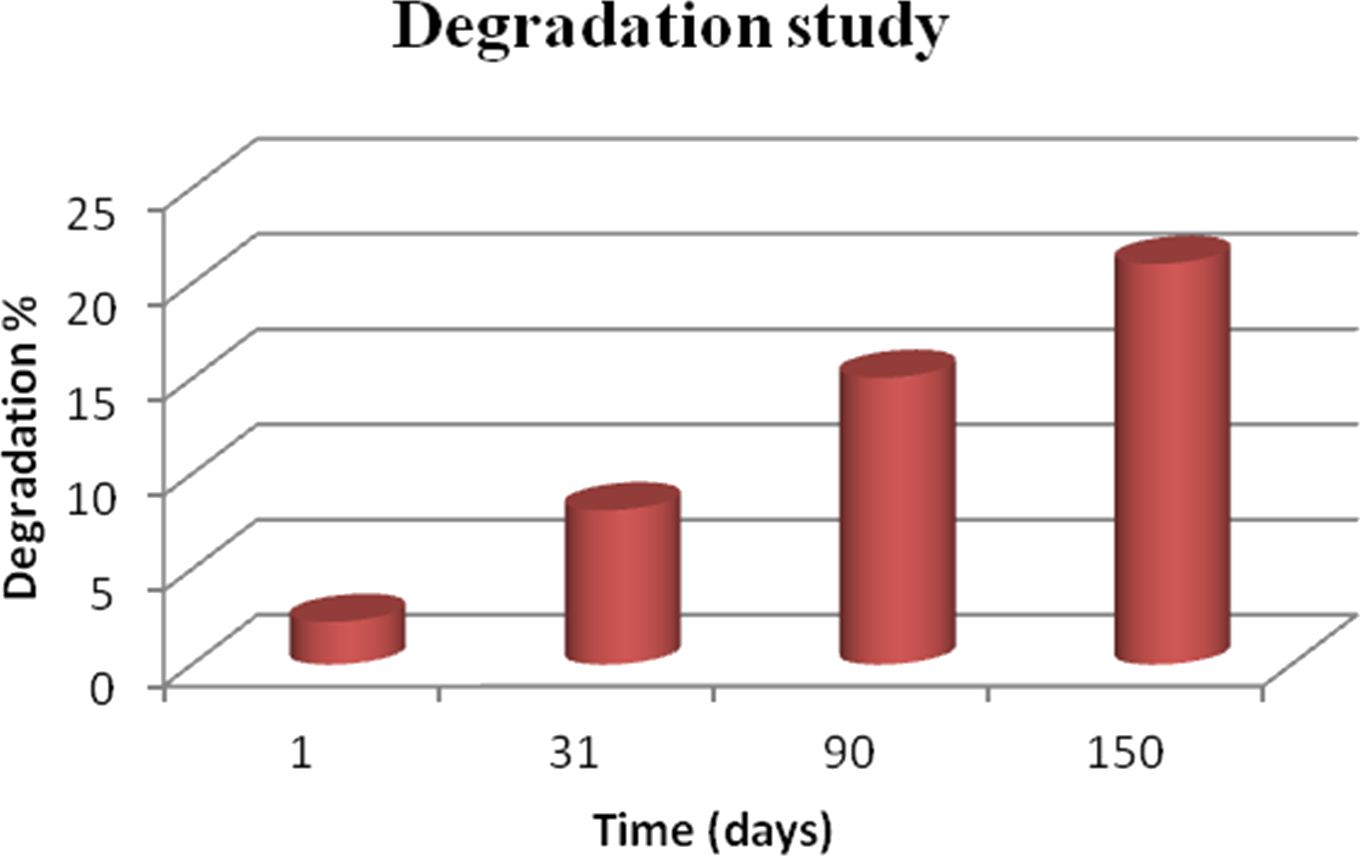
In vitro degradation of CA scaffolds loaded with dexamethasone as function of the time.

A degradation study of CA:dexam scaffolds in DMEM solution at 37 °C over a period of 5 months was determined. It showed a change of 21% of the polymer scaffold mass (Figure 5). This degradation rate is even slower than that of drug-free CA scaffolds. It is obvious that the presence of the hydrophobic dexamethasone as well as the size of the CA:dexam fibers, which was larger than that of the pure CA fibers, were critical parameters. This led to a decrease in the active surface of the fibers which in turn reduced the hydrolysis resulting in a slower degradation rate of the CA:dexam fibers.

**Figure 6:**
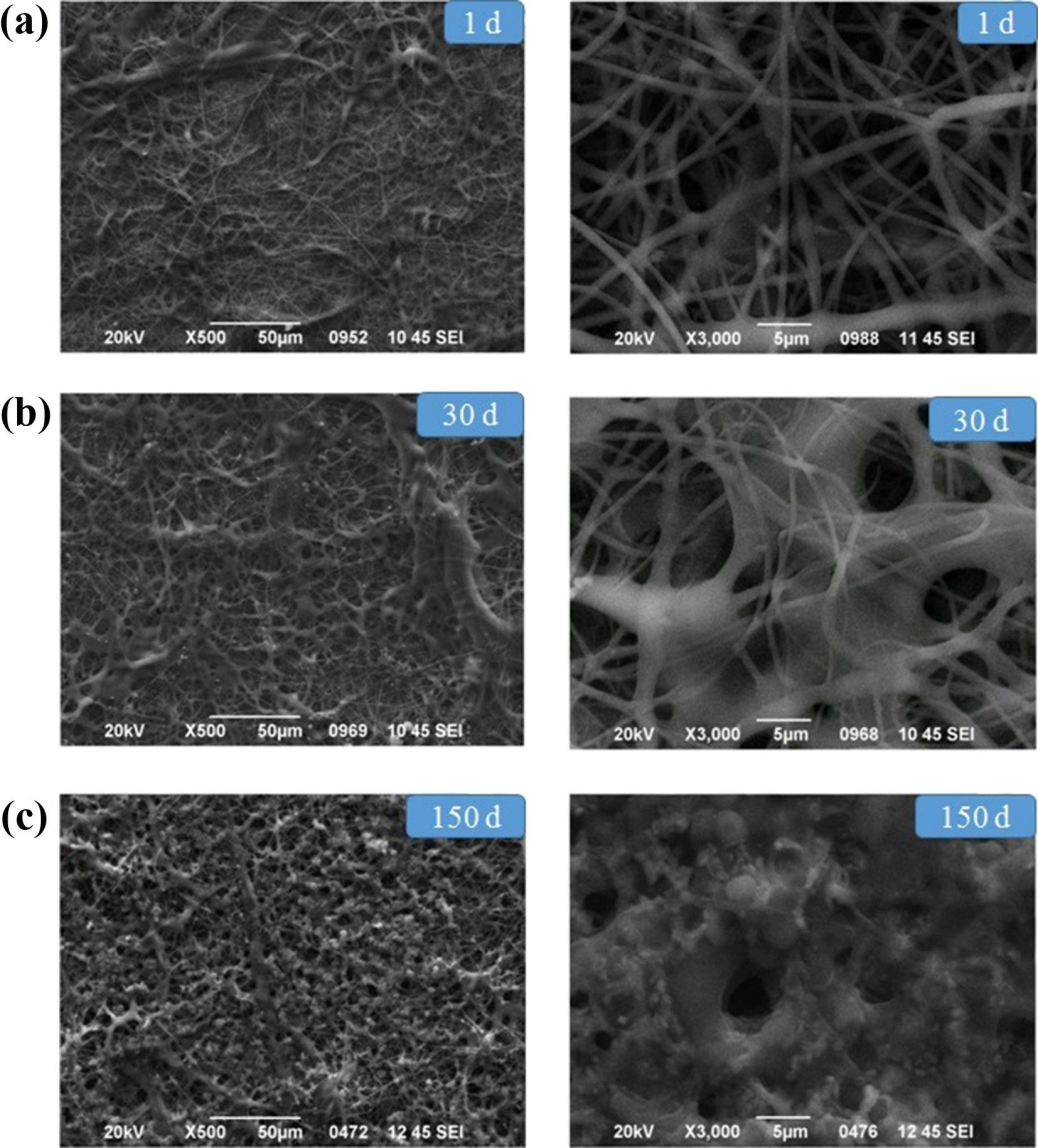
Representative SEM micrographs of CA scaffolds (a) before degradation on the 1st day, (b) after degradation in vitro for 30 days, (c) after degradation in vitro for 150 days. The mean diameters of the CA fibers after day 1 and day 30 were 1756 and 4078 nm, respectively.

The degradation of CA:dexam fibers after 1, 30 and 150 days was also examined by using SEM and the results are presented in Figure 6. In addition, 20 fibers were randomly selected from the images at the highest magnification (3000×) and their diameter was determined by using the ImageJ software. Again, from day 1 to day 30 the average fiber diameter increased due to fiber swelling, from 1756 to 4078 nm. After 150 days the fibers had melted to a great extent, making the determination of their diameter impossible.

### Dexamethasone-loaded scaffolds

#### In vitro release of dexamethasone

**Figure 7:**
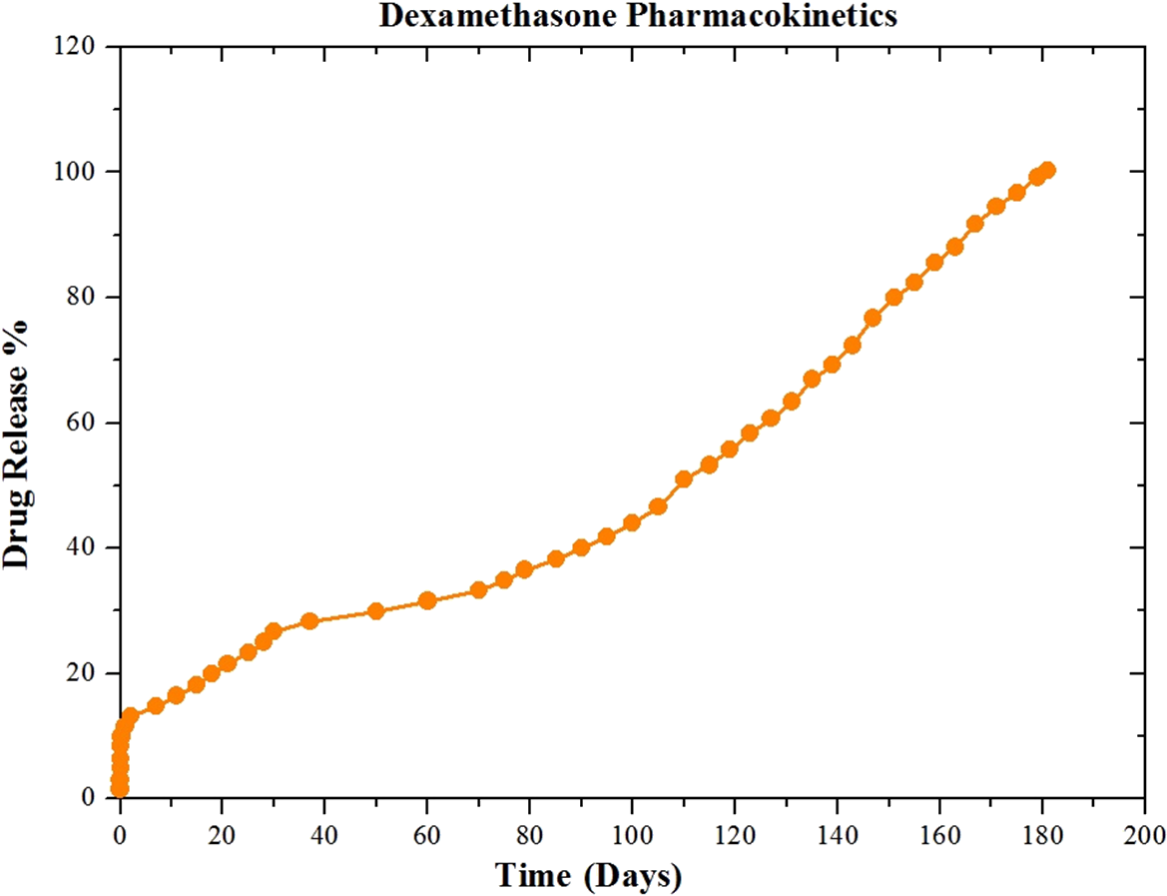
In vitro release of dexamethasone.

The in vitro release of dexamethasone is presented in Figure 7. The release of dexamethasone from the scaffolds exhibits a biphasic release pattern with an initial burst on day 1, in which 11.6% of the dexamethasone was released. This is probably due to the hydrophobicity of dexamethasone. When a hydrophobic drug forms a fibrous matrix with a polymer, a low burst release is observed. The second stage is the decay of the polymeric matrix during which the degradation of the polymer is most important. The drug is released as the CA fibers melt. Generally, a slow and controlled release was observed up to six months, reaching a release rate of approximately 96.8% after 175 days. The release of dexamethasone from the scaffold was completed after 181 days.

The burst release of the drug delivery system was very low (11.6%) and, consequently, this nanoplatform will not release the necessary amount of the drug to merge the immunosuppressive effect over a time scale of 181 days. In the same context, the choice of the CA scaffold, the degradation pattern of which is appropriate for this application, contributed positively to the anti-inflammatory action of dexamethasone and not to its immunosuppressive action.

#### Cytocompatibility behavior

**Figure 8:**
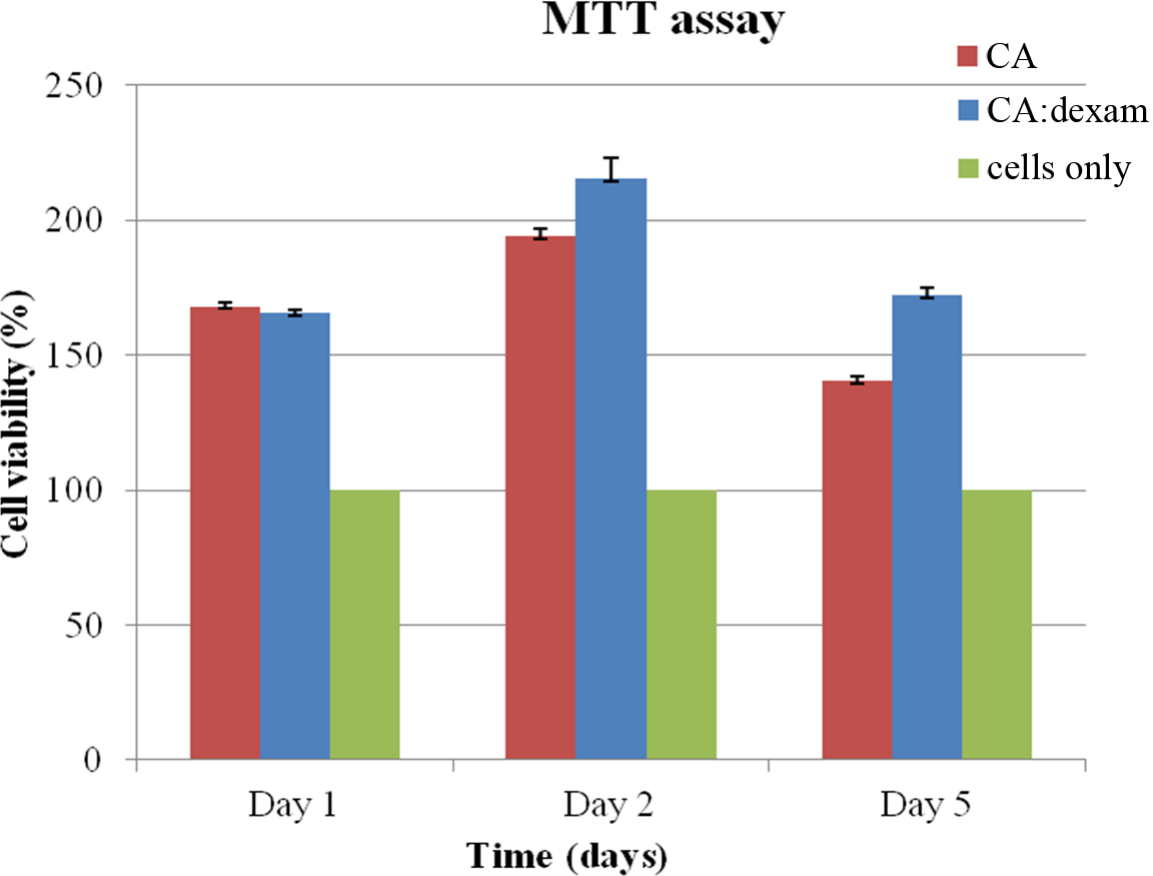
MTT assay of L929 cells with the examined scaffolds after 1, 2 and 5 days.

The MTT assay shows the quantitative analysis of cell proliferation as a function of the time. The MTT results of the samples in contact with L929 cells are shown in Figure 8. The absorbance values of both scaffolds on the first day compared to the control group (cells only) were sufficiently high, indicating the initial adhesion of the cells to the surface. Then, the cell population gradually increased until the second day, while a small reduction was observed on the fifth day, possibly due to the fact that the cells were developed and multiplied on the surfaces at such a rapid rate that there was no further space. Therefore, they began to overflow in order to survive and descended under the specimens and into the well-plates where they were located, so as to favor and continue their growth in a place where there was still nutritional material (DMEM). It has been reported that dexamethasone inhibits the proliferation rate of fibroblast cells and induces apoptosis. Glucocorticoids possibly induce the synthesis of some proteins that are able to compromise multiple systems and inhibit growth. This might be due to the modification of other proteins that play crucial role in different cellular events. Nevertheless, cultured fibroblasts respond to glucocorticoids either with a positive or negative growth effect [25]. According to the results, the cells recognized their new microenvironment and proliferated, as all values of the fibrous scaffolds were above those of the control sample, proving that these nanoplatforms are cytocompatible.

**Figure 9:**
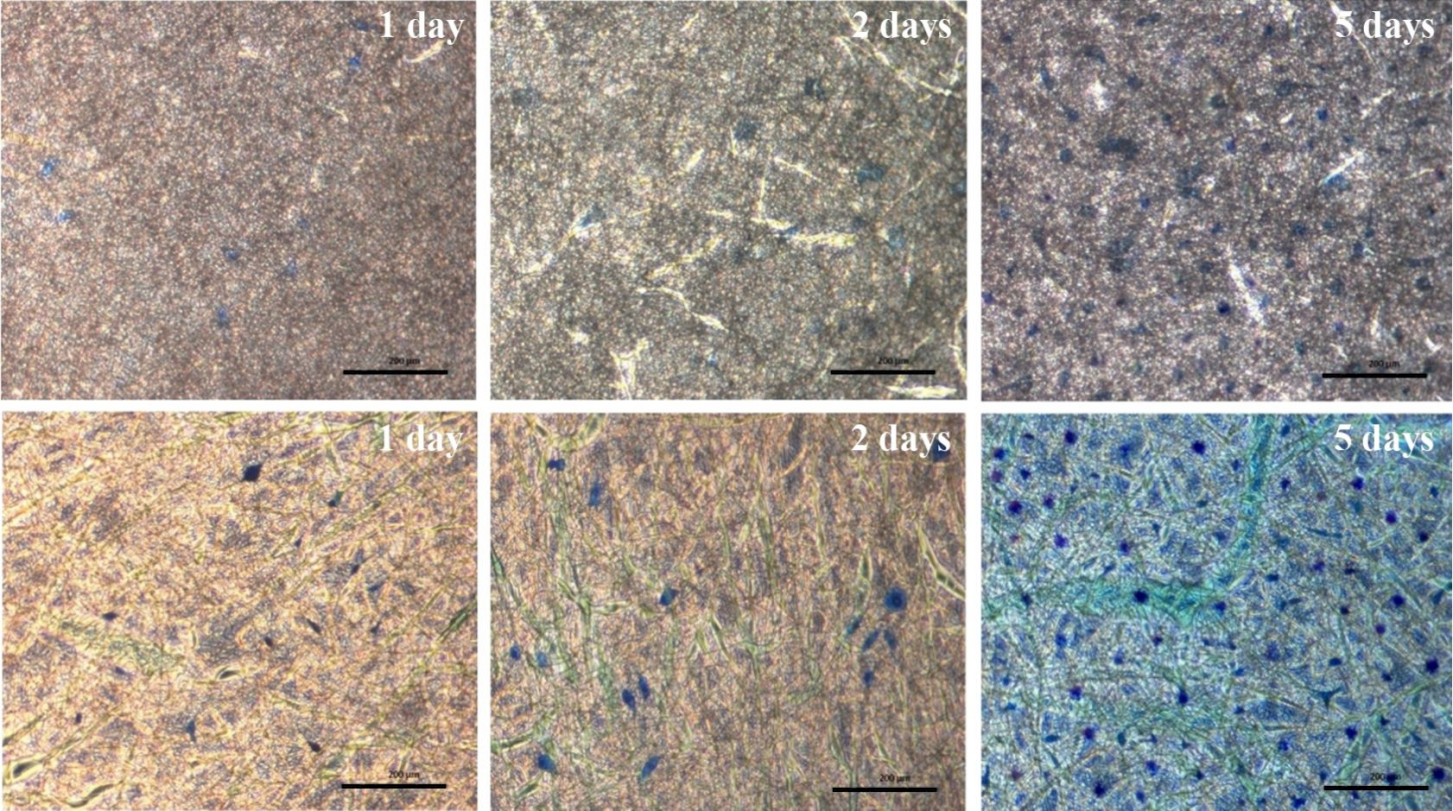
Optical microscopy images of the cell morphology on the examined scaffolds. The three pictures in the upper row display the CA scaffolds, while the three pictures in the lower row indicate the CA:dexam scaffolds after the 1st, 2nd and 5th day, respectively (scale bars: 200 μm).

In order to confirm the above results of the MTT measurements, microscopic observation of the nuclei of cells grown at the surface of the CA and CA:dexam scaffolds after methylene blue staining was also performed. Figure 9 shows that the proliferation of cells for both scaffolds is gradually increased over time. On day 5, the cell population was more pronounced and had almost covered the entire surface of the scaffolds indicating that the scaffolds exhibited good cytocompatibility.

**Figure 10:**
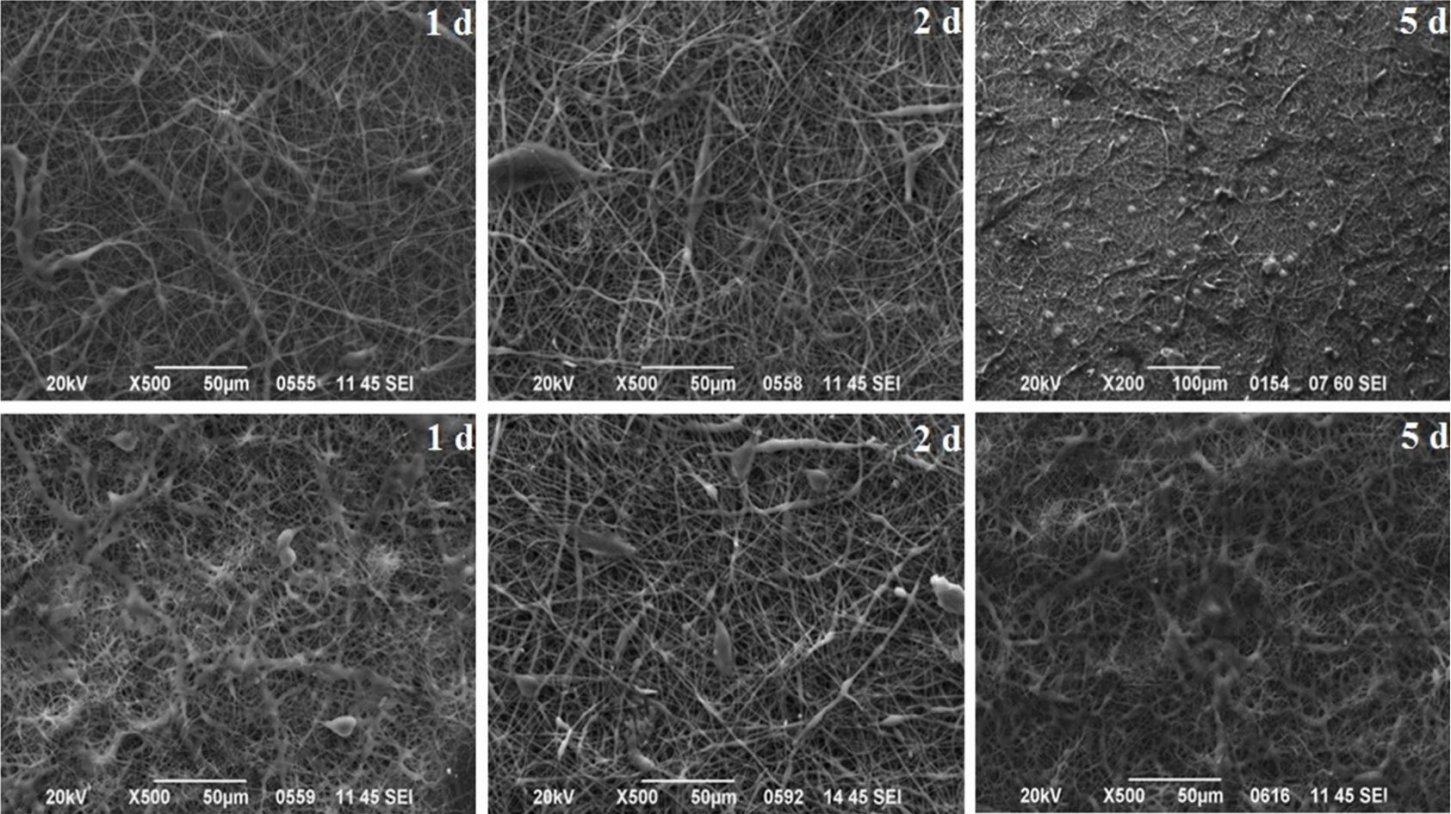
SEM micrograph of L929 cells on the examined scaffolds. The three pictures in the top row display the CA scaffolds, while the three pictures in the bottom row indicate the CA:dexam scaffolds after the 1st, 2nd and 5th day, respectively.

A small area of the scaffold surface was isolated through SEM in order to examine the behavior of the cells as a function of the time. It is clear from Figure 10 that the cells on both scaffolds recognized their microenvironment and proliferated. The cells also began to spread, indicating that the scaffolds are cytocompatible. Moreover, on the dexamethasone-loaded scaffolds, the cell population was larger than on the drug-free CA scaffolds. This could be attributed to the higher roughness created of the drug-loaded scaffold, as measured with AFM, which created a particularly favorable environment for the rapid growth and proliferation of cells on its surface, as well as to the reasons mentioned before in the discussion of the ΜΤΤ assay.

**Figure 11:**
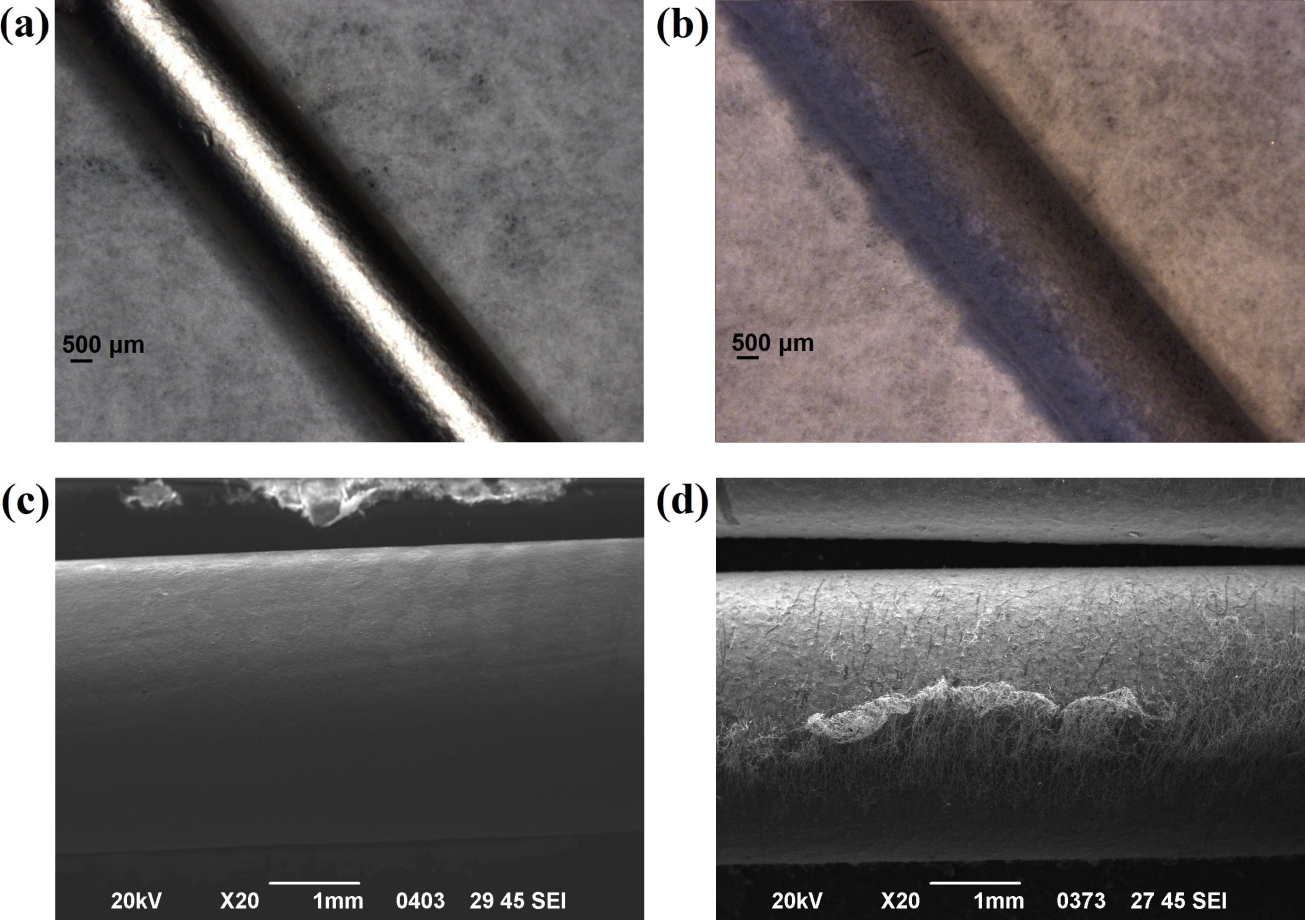
Keithley microscopy images of (a) a blank orthopedic pin, (b) a pin coated with CA:dexam fibers and SEM micrographs of (c) a blank orthopedic pin, (d) a pin coated with CA:dexam fibers.

These results were quite promising. Hence, this nanoplatform was also developed as coating onto an orthopedic pin for further in vivo testing (Figure 11).

## Conclusion

Our findings show that a good fiber-based morphology of cellulose acetate scaffolds was achieved with larger diameters of the drug-loaded scaffold fibers. AFM and SEM measurements validated the successful fabrication of those structures. The release of dexamethasone exhibited a biphasic release pattern, with an initial burst on the first day, followed by a slow and controlled release. In vitro degradation studies were performed and verified that the degradation rate of the drug-loaded scaffolds was even slower than that of the pure CA scaffold. The presence of the drug delayed the decay of polymeric matrix. Finally, this study demonstrated that dexamethasone-loaded electrospun CA scaffolds provide a environment in which cells (standard immortalized fibroblasts L929) grow and proliferate, making this nanoplatform cytocompatible. In future studies, the interaction with musculoskeletal tissues will be examined with other cell lines such as mesenchymal stem cells (MSCs) or bone marrow stromal cells (BMSC) in order to evaluate the tissue specific response to our scaffolds. This cytocompatible nanoplatform might be a suitable and helpful candidate to reduce implant-associated acute inflammations and to impede implant failure.
